# Pollen Diet—Properties and Impact on a Bee Colony

**DOI:** 10.3390/insects12090798

**Published:** 2021-09-06

**Authors:** Maciej Sylwester Bryś, Patrycja Skowronek, Aneta Strachecka

**Affiliations:** Department of Invertebrate Ecophysiology and Experimental Biology, University of Life Sciences in Lublin, Akademicka 13, 20-950 Lublin, Poland; patrycja.skowronek@up.lublin.pl

**Keywords:** nutrition pollen, monofloral diet, multifloral diet, *Apis mellifera*

## Abstract

**Simple Summary:**

In order to study the effects of malnutrition in bees, attention should be drawn to the dietary composition. A pollen diet plays an important role in the life of bees. A well-balanced diet influences the development of the larvae as well as the physiology, biochemistry, immunity and histology of the workers.

**Abstract:**

Diet is an important factor in the proper development of the individual and the entire colony. A pollen diet affects honey bees in a number of ways. It can stimulate the number and type of hemocytes, the total number of proteins, carbohydrates and lipids, affect the histology of the middle intestine, and ensure the correct ontogenesis of the larvae. Moreover, selected single-flower diets can stimulate the development of the pharyngeal glands that produce royal jelly, thus conditioning the development of secretory immunity. Selected single-species pollen may also increase the phenol oxidase concentration, which contributes to the humoral response. A honey bee diet based on multi-flower pollen is more desirable than a mono-flower diet, but must be properly balanced.

## 1. Introduction

Honey bees (*Apis mellifera* L.) play an important role in the environment as leading pollinators. Workers of this species have an impact on the pollination of agricultural crops and wild vegetation [1]. The proper development of a colony is conditioned by the availability of nectar and flower pollen. The growth and development of the organism may be limited by unbalanced stoichiometry, including incorrect proportions of chemical elements in food [2]. Insects need various ingredients to meet their nutritional requirements [3]. Proteins, carbohydrates, minerals, lipids, vitamins, and water are the staple ingredients of their diet.

Nutritional deficiencies, parasitic mites, numerous diseases and declines in populations due to colony collapse disorders (CCD) are contributing to losses worldwide [4,5,6]. The period of hunger, characterized by the lack of food, determines the consumption of supplies. Nectar and pollen depletion negatively affects brood rearing, honey production, and overall colony growth and development [7]. The availability of nutrients in the environment does not always match the needs of a colony. The lack of one or more components can lead to population reduction, shortened life expectancy, and disease susceptibility by weakening bee immunity [3]. Decreasing plant diversity may be a key factor driving the decline in bees [2]. Observing the availability and flow of nutrients in the environment can help solve the present problem of pollinating insects [7]. Moreover, bee losses are caused by agricultural chemicals [8,9], environmental variability, malnutrition [10], and the synergistic effects of some or many of the above factors [10,11].

In the last decade, destruction and fragmentation of habitats turned out to be a severe problem for pollinators, which resulted in reduced availability of bees’ flower resources [12]. Moreover, monocultural plantings reduced the diversity of flowering plant species within the radius of bees’ feeding distance [13,14]. The mass-flowering monocultures may provide only transient forage for bees [15,16]. However, such a loss of resource diversity can lead to sub-optimal bee nutrition, resulting in a weakened bee immune system and poor overall health [17,18].

Beekeepers, apart from the stationary apiaries, have so-called migratory apiaries to avoid a period of hunger and/or a reduction in seasonal survival of bees during severe pollen decline (e.g., between rapeseed (*Brassica napus*) and sunflower, (*Helianthus annuus*) between mass flowering in intensive agriculture systems. They move their hives to places where they can obtain more honey while providing insects with access to a diverse base of benefits. The economy of migration, however, is associated with high costs, additional work, and the transmission of bee parasites and pathogens over long distances. It is estimated that 40% of bee colonies continue to die of hunger during this migration [6]. As a result, beekeepers are forced to use artificial seasonal bee feeding to sustain egg laying, brood development, and basic population activities [7].

## 2. Flower Pollen 

Many plant species produce pollen that is poor in proteins, e.g., protein-poor pollens are produced by conifers and grasses, which are non-nectar-yielding plants [19]. The protein content in the pollen of various plants is highly variable, ranging from 2.5% to 61%, with an average of 25–45% [1,20]. The lowest protein content is found in Cactaceae and Onagraceae plants (15–25% of protein), while Melastomataceae, Cochlospermataceae and Solanaceae are the richest in protein (over 51%) [19].

Pollen of the same type of plant (e.g., *Rubus* type, *Taraxacum* type, *Prunus* type) shows similar protein profiles. Bee pollen is known to contain an estimated 5530 mg/kg of potassium-, 4600 mg/kg of phosphorus-, 2378 mg/kg of sulfur-, 1146 mg/kg of calcium-, 716 mg/kg of magnesium-, and sodium, iron, zinc, manganese and copper below 100 mg/kg [21]. The nutritional content in pollen differs from plant to plant. Bee bread is a mixture of honey and bee salivary enzymes [22]. All these compounds contained in pollen determine the antimicrobial protection of the body. It is believed that ten amino acids found in pollen are essential for the proper functioning of the honey bee. These amino acids are: threonine, valine, methionine, isoleucine, leucine, phenylalanine, histidine, lysine, arginine and tryptophan [21,23]. Glycine, proline, and serine are not as important as other amino acids. The nutritional value of pollen is reduced when inadequate amounts of essential amino acids are present [24]. Among 182 pollen samples tested, only 66 of them had isoleucine levels below the desired level [21]. Stored pollen showed lower nutritional value. After one year, pollen loses its stimulating properties by 76% [19]. Low-protein pollen exposes bees to severe amino acid deficiencies [20].

## 3. Digestion of Pollen

The digestive system of honey bees consists of three sections: the anterior, midgut and posterior intestine [25]. The anterior and posterior intestines are of ectodermal origin and are lined with a chitinous cuticle that protects them from pathogens [25,26,27]. The midgut digestive tract is responsible for digestive processes and food absorption [28]. This section should be brown in color and characterized by a strongly undulating surface [29].

**Figure 1:**
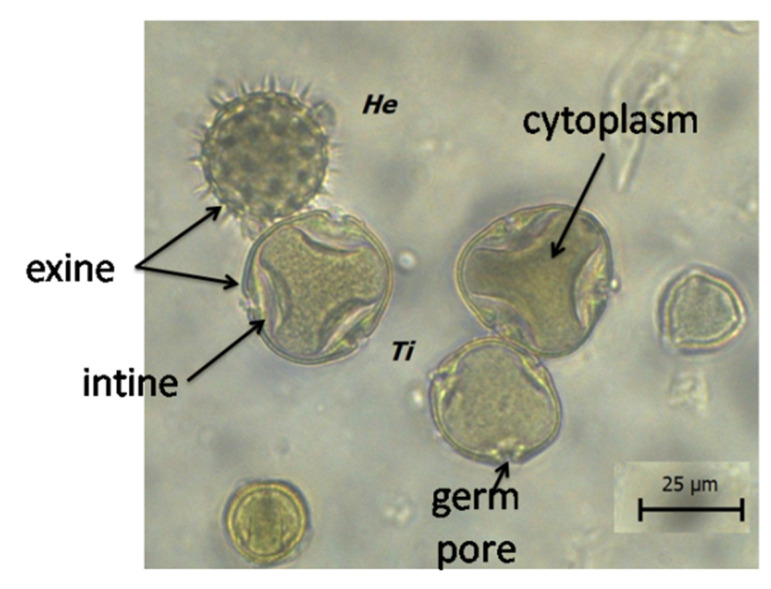
Pollen grain, He—Helianthus type, Ti—Tilia.

**Figure 2:**
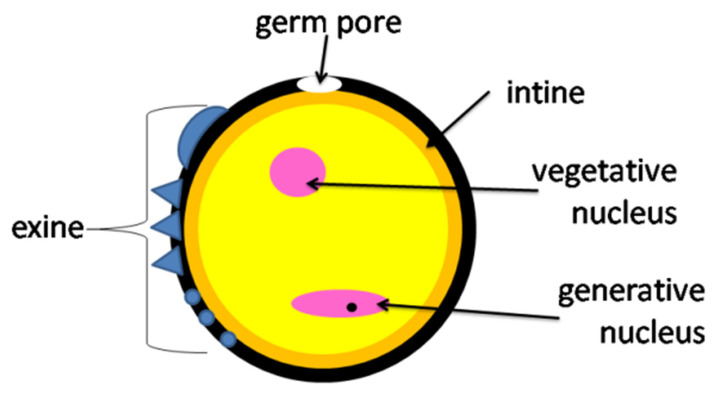
Structure of a pollen grain.

Digestion of pollen grains takes place in the midgut intestine [18]. The cell walls of pollen grains are resistant to digestive enzymes, thus it is common for pollen grains to pass through the digestive tract and remain undigested [20]. The digestion of pollen grains may vary between species. Crailsheim et al. [30] reported that the percentage of digested grains isolated from a bee rectum was higher for *Castanea* than *Trifolium* pollen. The authors conclude that the differences in pollen digestibility are due to the structure of the pollen cell walls. The outer wall of the pollen grain (exine) is composed of lipids, proteins, and sugars, while the inner layer (intine) is composed of cellulose and pectin [1] (Figure 1 and Figure 2). Insects must damage the cell wall to enter the nutrient-rich cytoplasm [1,10] and they use various mechanisms for this purpose: (1) mechanical grinding through the mouth piece; (2) biochemical action with the use of digestive enzymes; (3) osmotic shock [20].

Pollen protein digestion and absorption are determined by the animal’s species, age, diet (nutrient content and proportion), environmental factors, and feeding technology [10,20,31].

Proteases are enzymes responsible for digesting protein in the honey bee’s digestive tract. Many proteases in the digestive tract of insects are analogs to trypsin and chymotrypsin in mammals [32]. Newly emerged bees have low proteolytic enzyme activities which increase with age. Proteolytic activities were the highest in six-day-old bees and are associated with the development of the midgut. Moreover, the thickness of the peritrophic membrane (PM) is influenced by pollen proteins [31]. Wang et al. [31] noted that the PM of bees fed with camelia pollen was much thinner than that of those maintained on rape pollen diets.

Pollen consumption stimulates the secretion of glycosidic hydrolase (GH), which is accumulated in the middle and large intestine that harbors a core microbial environment composed of approximately 10^8^ bacterial cells. GH acts synergistically with the host’s digestive enzymes and participates in carbohydrate metabolism, aided by the gut microflora. As a result of pollen digestion, mono-, di- and oligosaccharides are released. The glycosidic linkage, inaccessible to the host enzyme, can be further cleaved by colon bacteria. The resulting aglycone can be metabolized by the host and by the microflora [33]. Not much is known about dietary indigestible carbohydrates, which are the main components of pollen biomass.

## 4. The Influence of the Pollen Diet on the Physiology and Histology of the Middle Intestine of a Honey Bee

A strong colony of 200,000 bees need at least 25 kg of pollen per year. This value may be underestimated as pollen consumption by bees resulting from beeswax production has not been taken into account [34]. The individual development of the honey bee will differ depending on the amount of available pollen, as well as on the protein composition of pollen, influencing, among other things, its physiology.

Worker bees use freshly collected pollen to feed the larvae. Young bees aged from three to six days consume the most pollen in spring. This period can extend up to nine days in the summer [19]. The larval development period is decisive for meeting the demand for all elements [2]. The pollen diet also affects the weight of the larvae. As reported by Tasei, and Aupinel [35], bumble bee larvae fed with multiflorous pollen were heavier than those administered monoflower pollen with increased protein content. When protein content in a diet is low, bees limit their brood rearing [18].

Frias et al. [1] investigated the effect of pollen quality by monitoring how pollen protein content and pollen diversity influence the physiology and health of adult bees in a queenless setting. It has been proven that there is a link between pollen nourishment and the activation of the ovaries in queenless workers when fed with high- or low-protein pollen. Bees kept on a diet with a lower protein/carbohydrate ratio lived longer, but those that were administered royal jelly diets had higher ovarian activation, which was due to the presence of other nutrients in royal jelly in addition to protein. In addition, the activation of the ovaries depends not only on the protein content of the pollen but also on other nutrients present in the pollen such as lipids and carbohydrates.

**Figure 3:**
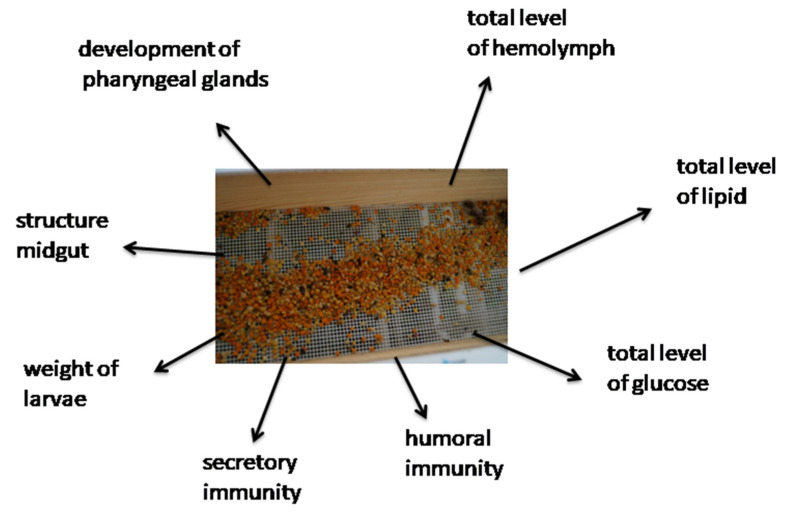
Influence of pollen diet on a honey bee.

The effect of a protein diet was investigated in the context of the development of the hypopharyngeal glands (HPG) in worker bees (Figure 3). This is an important factor as HPG secretes jelly, the glandular protein secretion that juveniles are fed by young bees [7]. Honey bee workers had the most developed glands and were fed with bee bread only, and then with yeast. Fresh pollen stimulates the development of the pharyngeal glands, while the stimulating effect of stored pollen is reduced by 76% [36]. The difference in the development of the pharyngeal gland was also observed to be affected by the composition of the pollen diet. Bees fed with *Rubus* pollen had more developed throat glands than those fed with *Cistus* and *Erica* pollen [7]. Omar et al. [37] checked the impact of monodiet on the development of HPG. They observed that the pollen of *Castanea* sp. and *Asparagus* sp. stimulated the development of HPG (Table 1).

Moreover, bee pollen contains proteins and amino acids that contribute to longevity [38]. Frias et al. [1] showed that brood rearing and colony life span is reduced if the bee’s access to pollen is hindered. In the absence of food, the gatherers die earlier than their sisters working in the hive. The pollen mix of the Asteraceae, Moraceae, and Myrtaceae families was prepared as the most common pollen types. Low survival rate of adult workers was observed with the pollen diet based on Asteraceae. This type of pollen was unpalatable to bees because consumption was negatively correlated with the percentage of Asteraceae in the mixture [1]. As reported by Roulston et al. [20], the protein content of pollen from the Asteraceae family is very low compared to other plants. This group of plants includes, for example, *Taraxacum officinalis* and *Helianthus annus*. The pollen of these plants is low in amino acids and a diet based on this pollen does not meet the nutritional needs of bees. Moreover, pollen from the Asteraceae family is characterized by a low digestion index [22]. A multi-flower diet is better than a diet based on a single species. *Brassica napus* pollen may be the exception which is good for insects, because this pollen is highly nutritious to bees [39].

**Table 1:** Influence of pollen diet on the hypopharyngeal glands and hemolymph (based on the literature [7,20,37,40]).

Type of Pollen/Parameter	*Rubus*	*Castanea* sp.	*Asparagus* sp.	Asteraceae	*Zea mays*	*Trifolium* sp.	*Vicia faba*	*Phoenix* *dactylifera*	*Erica*
pharyngeal glands	↑	↑	↑						
survivability				↓					
plasmocyte					↑	↓			
coagulocyte						↑			
prohemocyte							↑		
oenocitoid							↑		
binucleated cells								↑	
total protein					~		~	~	
glucose					↑				
lipids					↑	↑	↑	↑	
phenol oxidase (PO)									↑

↑ increase, ↓ decrease, ~ not much difference.

The lack of dietary diversity alone is not the cause of massive bee extinction (CCD), but the diet increases susceptibility to stressors including *Varroa destructor, Nosema ceranae, Nosema apis*, and pesticides. Such stress may also be related to the non-random selection of plant species by bees [41]. Nutritional stress translates into brood count and the physiological development of insects.

Feeding bees with pollen also affects the histology of the midgut. The eight-day diet based on a pollen substitute resulted in a higher midgut epithelium, granular cytoplasm, slightly dilated intestinal lumen, and the rhabdorium and regenerative crypts unchanged [42]. Increased thickness of the peritrophic membranes enhances digestive efficiency because the consumed food can stay longer in the intestine and can be utilised better by the organism. The expansion of the intestinal lumen in bees, in particular in those which were fed bee bread, indicates flatulence and/or the presence in the feed of substances causing gas secretion and/or the presence of mineral compounds [28]. The *Camelia* pollen diet resulted in tighter distribution and smaller diameter of crypts in the epithelium of the middle intestine [20]. Wang et al. [31] suggested that diet effects are the primary factor that influenced midgut development. By influencing midgut development, the diets cause discrepancies in digestive and absorptive functions, which are ultimately reflected by differences in protein digestion.

## 5. The Influence of the Pollen Diet on the Composition of the Hemolymph

The insect hemolymph is an analogue of the blood of vertebrates and is responsible for the supply of nutrients between the tissues and organs [43]. In addition, it plays an important role in the storage of nutrients and provides defense against microorganisms [40]. The hemolymph contains water, inorganic salts, proteins, carbohydrates, hormones, lipids, free amino acids, and hemocytes [43]. The honey bee has an open circulatory system and numerous hemocytes contained in the hemolymph. Hemocytes have variable morphology and perform different functions, but are related to defense responses. They have the ability to phagocytosis small particles such as bacteria, fungal spores, and some parasites [40].

Monofloral diets were compared for their effects on the hemolymph composition (Table 1). In the group of bees fed with corn pollen (*Zea mays*), a high percentage of plasmocytes was found, while the clover diet (*Trifolium alexandrinum*) caused their decrease in relation to other hemocytes (e.g., coagulocyte cells, prohemocytes and oenocytes). Moreover, clover pollen determined the increased content of coagulocyte cells, which was the lowest in the bees fed with corn pollen. The highest concentrations of prohemocytes and oenocytes were found after feeding with broad bean pollen (*Vicia faba*). The pollen diet based on the date palm (*Phoenix* *dactylifera*) resulted in a significant increase in binuclear cells compared to the control sample [40]. The present research proves that a single-species pollen diet influences the differences in the content of individual hemocytes.

Bees obtain carbohydrates from nectar and protein from pollen [1,3]. Foraging bees are not able to assess the protein content of the harvested material, so pollen selection is a food lottery. For example, workers also collect sawdust, coal dust, and wood humus [44]. The consumption of pollen is reflected in a change in the protein concentration in the hemolymph, which varies with developmental stages [43]. Moreover, the total level of proteins in the hemolymph of workers fed a protein diet is higher than that of workers fed with sugar syrup [45]. As a result of pollen feeding, the content of storage proteins such as vitellogenin (Vg), yolk protein, and hexamerin (Hex) increases. These proteins are synthesized from the fat body and secreted into the hemolymph [1]. Moreover, Vg regulates a variety of physiological aspects, including behavioral development, life expectancy, and immunity [46]. For every 10 g of protein, approximately 48 g of pollen is required, containing 30% crude white [20]. Date palm pollen increased the protein concentration in the hemolymph by one unit expressed in mg/mL. Protein concentrations in hemolymph were approximately of 8 mg/mL after ingesting pollen from faba bean, maize, or clover [42]. Almeida-Dias et al. [45] checked the attractiveness of the pollen diet after pollen fermentation. Bees were more likely to take fermented pollen, and this translated into a higher level of proteins in the insects’ hemolymph [45].

The main source of energy for social insects are sugars, mainly glucose. Research by El Mohandes et al. [40] proved that the type of pollen diet influences the glucose content in the insect’s hemolymph. The highest value of glucose was recorded for the feeding with maize pollen (7.38 mg/mL), and the lowest value for date palm pollen (6.86 mg/mL).

Lipids play an important role in various physiological/biochemical processes. They are a source of energy for reproduction, growth, and development. Lipids are universally the primary component of the lipid bilayer of the cell membrane and are stored as energy reserves in the fat body and intracellular lipid globules. *A. mellifera* obtains lipids from pollen. Low diet diversity can expose bees to nutritional stress. It has been proven that adequate access to lipids determines the development of brood and affects the longevity and body structure of insects [46,47]. The pollen diet also influences the lipid content of the hemolymph. The bees from the control group, fed with soy flour based artificial diet, had the highest lipid concentration of 50 mg/mL. A similar lipid result was caused by the faba bean pollen, maize pollen, and clover pollen diets (over 49.00 mg/mL). The lowest concentration of lipids (48.36 mg/mL) in the hemolymph was determined for the date palm pollen diet [40]. The pollen diet influences the composition of the hemolymph, including the diversity of hemocytes, the content of glucose, fats and proteins, and the histology of the middle intestine. These elements influence the honey bee’s immune mechanisms in the fight against pathogens. Incorrect pollen diet caused by fragmentation, destruction of natural habitats, and monocultures may weaken the bee colony.

## 6. The Influence of the Pollen Diet on Immunity

The functioning of the insect immune system requires a great deal of energy as honey bee immunity is complex. *A. mellifera* has developed mechanisms of individual and collective resistance [48]. Moreover, there is an innate and induced immune reaction in insects [49]. Individual immunity is divided into cellular (phagocytosis, encapsulation, and nodulation) and humoral (lysozyme, hemagglutinins, humoral encapsulation, phenol oxidase) [27,50].

An improperly balanced diet may weaken the functioning of the immune system and increase the susceptibility of workers to diseases [38]. Even a small addition of the adequate amount of good-quality protein to the diet of insects may positively influence the immune system of pollinators, but not much is known about this [38].

Phenoloxidase (PO) plays an important role in various physiological and humoral immunity. Di Pasquale et al. [7] showed that the level of PO in the insect’s hemolymph can be regulated by the quality of the diet. PO influences the synthesis of melanin, which is a quinone polymer containing nitrogen [7]. However, Alaux et al. [38] found no dietary effect on the PO content of the hemolymph. On the other hand, Di Pasquale et al. [7] proved that *Erica*’s pollen increased the level of PO. These authors suggest that more research is needed to test the effect of the pollen diet on PO.

Secretory immunity, which is characterized by the presence of antimicrobial compounds in bee secretions and products [27], plays an important role in feeding larvae and young workers. For example, royal jelly reduces the population of bacteria by getting into the digestive tract of larvae with food. It is bactericidal and bacteriostatic. Honey contains lysozyme, phenolic acids, and flavonoids [51]. Antibacterial properties of honey largely depend on the accumulation of hydrogen peroxide (H_2_O_2_), which is generated by glucose oxidase (GOX) [52].

## 7. Conclusions

One of the reasons for declining bee colonies is a non-stoichiometrically balanced diet. In order to prevent this from happening, it is necessary to understand the influence of diet on the physiology, biochemistry, and immune system of these insects. In order not to draw erroneous conclusions, it is necessary to conduct a thorough study that will indicate the correct diet for these insects. While single species pollen has specific benefits, bees require pollen from diverse sources to maintain a healthy physiology and hive. Multipollen diet should be offered to the bees to derive requisite benefits and at the same time be secure of the colony requirements. The literature describes a single-species diet in two ways. On the one hand, there are many negative reports on the impact of monocultures from which such pollen that originates from monoculture influence the health, functioning and survival of bees. On the other hand, monoflower pollen is presented as having positive properties. Therefore, it should not be assumed that a single-species diet is nutritionally inadequate, as it has been shown that selected pollen has a positive effect on some parameters. Multifaceted understanding of the influence of one type of pollen on a given parameter will make it possible to compose an ideal diet for a honey bee. Ultimately, it will be a multi-pollen diet, but selected in such a way as to effectively condition the appropriate response. Thus, it will be possible to adjust the diet as appropriate according to the requirements of bee colonies and reduce starvation stress.
